# Measurement properties and performance of an eight-minute submaximal treadmill test in patients with juvenile idiopathic arthritis: a controlled study

**DOI:** 10.1186/s12969-019-0316-7

**Published:** 2019-04-08

**Authors:** Kristine Risum, Elisabeth Edvardsen, Anne M. Selvaag, Hanne Dagfinrud, Helga Sanner

**Affiliations:** 1 Division of Orthopaedic Surgery, Section for Orthopaedic Rehabilitation, Oslo University Hospital, Rikshospitalet, Postboks 4950 Nydalen, 0424 Oslo, Norway; 2 Department of Health Sciences, Institute of Health and Society, Oslo, Faculty of Medicine, University of Oslo, Oslo, Norway; 3 Department of Pulmonary Medicine, Oslo University Hospital, Ullevål, Oslo, Norway; 4 Department of Physical Performance, Norwegian School of Sports Sciences, Oslo, Norway; 5 Department of Rheumatology, Oslo University Hospital, Rikshospitalet, Oslo, Norway; 6 National Advisory Unit on Rehabilitation in Rheumatology, Diakonhjemmet Hospital, Oslo, Norway; 7 Norwegian National Advisory Unit on Rheumatic Diseases in Children and Adolescents, Oslo University Hospital, Rikshospitalet, Oslo, Norway; 8 Bjørknes University College, Oslo, Norway

**Keywords:** Juvenile idiopathic arthritis, Cardiorespiratory fitness, Exercise testing, Validity, Reliability, Measurement properties

## Abstract

**Background:**

Poor cardiorespiratory fitness is previously reported in patients with juvenile idiopathic arthritis (JIA) measured both by maximal and submaximal exercise tests, but a submaximal exercise test with acceptable measurement properties is currently lacking for both clinical and research purposes in this patient population. The objectives of this study were to evaluate the measurement properties and performance of a submaximal treadmill test in patients with JIA, and to compare the results with those obtained in controls.

**Methods:**

Fifty-nine patients (50 girls), aged 10–16 years, with oligo- (*n* = 30) and polyarticular (*n* = 29) JIA, and 59 age- and sex-matched controls performed an eight-minute submaximal treadmill test for estimating peak oxygen uptake (VO_2peak_) followed by a maximal treadmill test measuring VO_2peak_ directly. During the submaximal treadmill test, the study participants walked with no inclination at a speed between 3.2–7.2 km/h for four minutes, and then continued to walk at the same speed for four minutes with five % inclination. VO_2peak_ was directly measured during a continuous graded exercise test on treadmill until exhaustion. Thirty-seven patients participated in the evaluation of the reliability. Criterion validity and reliability were evaluated with interclass correlation coefficient (ICC); measurement errors by Bland-Altman plot, standard error of measurement and smallest detectable change.

**Results:**

In patients with JIA, the ICC (95% CI) for criterion validity was acceptable at group level 0.71 (0.51, 0.82), but not at individual level. The test-retest reliability and inter-rater reliability were acceptable at individual (0.84 (0.71, 0.91) and 0.92 (0.83, 0.96), respectively) and group levels (0.91 (0.83, 0.96) and 0.96 (0.91, 0.98), respectively). The measurement errors (for test-retest reliability/inter-rater reliability) were large. Bland-Altman plots showed no systematic differences, but a large variability for both the validity and reliability. The performance of and estimated VO_2peak_ from the submaximal test were not associated with disease variables and were comparable between patients and controls.

**Conclusion:**

The submaximal treadmill test is valid for use in patients with JIA on group level, but not on individual level. The reliability is acceptable. Due to large measurement errors, the submaximal treadmill test is not optimal for use in daily clinical practice to estimate VO_2peak_ in individual patients.

## Background

Juvenile idiopathic arthritis (JIA) can affect physical function and cardiorespiratory fitness (CRF). CRF is important for general health, and high CRF has been shown to decrease cardiovascular disease in general pediatric and adult populations [1–3]. Previous studies have shown that patients with JIA have poor CRF measured with both maximal and submaximal exercise tests [4–6]. Contrary to these results, we have recently reported that patients with oligo- and polyarticular JIA diagnosed in the era of biologics have comparable levels of CRF as age- and sex-matched controls from the general population, measured directly as peak oxygen uptake (VO_2peak_) [7]. We believe our positive results may be explained by advances in the multidisciplinary management of JIA in the era of biologics, as well as differences in study populations. Importantly, 20–30% of both our patients with JIA and controls had poor CRF.

The gold standard method to measure CRF is through a cardiopulmonary exercise test (CPET) with direct measurement of VO_2peak_, using a treadmill or bicycle to maximal exhaustion [8]. However, a CPET is time consuming, requires advanced and expensive equipment in a laboratory setting and extensive experience to encourage individuals to achieve maximal effort. Furthermore, performing a maximal exercise test may be uncomfortable and unpleasant for patients.

In contrast, indirect submaximal tests do not require the individuals to exercise to exhaustion, are easier to perform, require less advanced equipment, and are therefore frequently used in research and clinical practice to measure CRF [9]. The disadvantage of submaximal tests is less precise measurements of CRF compared to direct measurement of VO_2peak_. Submaximal tests are usually developed to provide estimation of VO_2peak_ or to assess the distance covered in a given period of time or the time taken to cover a given distance. The most commonly used submaximal test in chronic pediatric conditions is probably the 6-min walk test (6MWT), even if the measurement properties vary largely among chronic pediatric conditions [10]. In JIA, the 6MWT has been suggested as a possible field test to measure walking ability, but is shown to be a poor predictor of VO_2peak_ [6, 11]. To the best of our knowledge, no submaximal walking tests aiming to estimate VO_2peak_ have been validated in pediatric populations.

An eight-minute submaximal treadmill test has been developed to estimate VO_2peak_ in healthy adults [12], and is proven valid for women with rheumatic diseases [13], who may experience similar symptoms as patients with pediatric rheumatic diseases. However, in healthy adults the test seems to either under- or overestimate VO_2peak_ depending on the chosen intensity [14]. The validity of this test is unknown for patients with JIA and healthy children. Also, knowledge about the reliability of the test is essential for both clinical practice and research purposes. Knowledge about how the performance of the test relate to disease variables is also warranted.

The objectives of the study were to evaluate the criterion validity and reliability of the eight-minute submaximal treadmill test in patients with JIA; also to investigate if the performance of the submaximal treadmill test is influenced by disease characteristics or differ from controls.

## Methods

### Study participants

This study is part of a larger study examining physical activity and physical fitness in patients with JIA diagnosed in the era of biologics [7, 15]. From January to August 2015, consecutive patients aged 10–16 years with polyarticular (extended oligoarthritis and polyarticular RF +/−) and oligoarticular JIA according to the ILAR criteria [16] with a planned routine follow-up at Oslo University Hospital (OUS) were recruited (JIA validity sample). We included these JIA categories to be able to compare homogenous JIA subgroups in the physical fitness and physical activity studies. Other inclusion criteria were disease duration > 6 months and a home address in the geographical area served by the South-Eastern Norway Regional Health Authority. Exclusion criteria for patients were comorbidities associated with, or potentially associated with, impaired cardiopulmonary fitness (e.g heart- or lung disease, severe orthopedic conditions or recent surgery) or inability to walk. In addition, age- and sex-matched controls from the general population (living in or nearby Oslo) were randomly selected from the National Registry, and were included from November 2015 to March 2016 (controls validity sample). Exclusion criteria for the controls were inflammatory rheumatic or autoimmune disease, severe heart or lung disease, or other diseases involving mobility problems.

To evaluate the reliability of the submaximal treadmill test, patients living in or nearby Oslo and patients with a planned follow-up at OUS within 4 weeks, also performed the submaximal treadmill test 1–4 weeks after the initial test (JIA reliability sample). In general, a sample size of 50 participants is considered to be adequate when assessing reliability and validity [17].

Our study was conducted in compliance with the Helsinki Declaration and all participants provided written informed consent (the children themselves if aged ≥16 years and the parents/guardians of children aged < 16 years together with the children’s assent). The study was approved by the Norwegian South East Regional Ethics Committee for Medical Research (2014/188).

### Assessment of demographic and disease-related variables

Height and bodyweight were measured to the nearest 0.1 cm using a stadiometer and 0.1 kg on a digital scale, respectively, with participants wearing light clothes and no shoes. Body mass index (BMI) was calculated. Waist circumference was measured at the midpoint between the bony markers of the ribs and the superior iliac crest in a standing position at the end of expiration with a measuring tape at the height of umbilicus to the nearest 0.1 cm. Current pain, pain and fatigue during the previous week were assessed by numeric rating scale (NRS) 0–10, where 0 = no pain/fatigue and 10 = worst possible pain/fatigue [18]. In patients, disease activity was assessed by the Juvenile Arthritis Disease Activity Score 71 (JADAS 71) [19]. The Wallace criteria were used to determine if patients had active disease or clinical inactive disease [20]. The Childhood Health Assessment Questionnaire (CHAQ) was used to measure functional disability [21, 22]. The patients completed the CHAQ themselves, with parental assistance if needed.

### Submaximal treadmill test

We used the submaximal treadmill test developed by Ebbeling et al. [12] to estimate VO_2peak_ (Technogym, Rimini, Italy). During the first four minutes of the test, the participant walked with no inclination at a speed between 3.2 km/h (2.0 mph) and 7.2 km/h (4.5 mph) corresponding to a heart rate (HR) between 50 to 70% of age-predicted peak HR (HR_peak_) of 220-age [23]. If possible, we aimed for a HR close to 70% of the predicted HR_peak_ and the speed was gradually increased until this intensity was reached. If a HR close to 70% of predicted HR_peak_ was not reached at the speed of 7.2 km/h (4.5 mph), the participant’s HR at this intensity was recorded. After four minutes, the treadmill elevation was then gradually increased (within 15–20 s) to five % for the next four minutes. HR was measured at the end of each stage with a heart rate monitor (Polar Sports Watch, Kempele, Finland). Participants rated their perceived exertion (RPE) using the Borg Scale _6–20_ [24] at three and eight minutes. The Borg Scale _6–20_ is a subjective measure of a person’s exertion during exercise, ranging from 6 to 20, where 6 = no exertion at all and 20 = maximal exertion. The HR and walking speed achieved after eight minutes of walking were then recorded for entry into the previously developed equation to estimate VO_2peak_ (mL∙kg^− 1^∙min^− 1^) based on the following equation [12]:

$$ \mathsf{15.1}+\left(\mathsf{21.8}\ \mathsf{x}\ \mathsf{speed}\ \left[\mathsf{mph}\right]\right)-\left(\mathsf{0.327}\ \mathsf{x}\ \mathsf{HR}\ \left[\mathsf{bpm}\right]\right)-\left(\mathsf{0.26}\ \mathsf{x}\ \mathsf{speed}\ \left[\mathsf{mph}\right]\ \mathsf{x}\ \mathsf{age}\ \left[\mathsf{yrs}\right]\right)+\left(\mathsf{0.00504}\ \mathsf{x}\ \mathsf{HR}\ \mathsf{x}\ \mathsf{age}\right)+\left(\mathsf{5.98}\ \mathsf{x}\ \mathsf{sex}\ \left[\mathsf{female}=\mathsf{0};\mathsf{male}=\mathsf{1}\right]\right) $$

We also recorded the total walking distance (m) the participants walked during the submaximal treadmill test. Evaluation of the submaximal treadmill performance included HR and RPE at three and eight minutes, speed and walking distance.

### Maximal treadmill test

CRF was directly measured as VO_2peak_ (mL∙kg^− 1^∙min^− 1^) during a maximal treadmill test (Woodway, Würzburg, Germany). The test protocol and procedure are described previously [7]. Briefly, gas exchange and ventilator variables were measured continuously breath-by-breath as the participants breathed into a two-way breathing mask (2700 series; Hans Rudolph, Inc., Shawnee KS, USA). The gas exchange variables were reported as 30 s averages using a gas analyzer (Vmax, SensorMedics, Yorba Linda, CA, USA). The highest achieved oxygen uptake averaged over a 30-s period was defined as VO_2peak_. The highest respiratory exchange ratio (RER) measured before or corresponding to the highest minute ventilation was reported. RER is the ratio between the VCO_2_ and VO_2_, and increases with exercise intensity. The HR was recorded every minute using Polar Sports Watch (Kempele, Finland) and the HR_peak_ was reported. The RPE was rated by Borg Scale _6–20_ [24], and the participants also gave reason for terminating the test. The test was terminated when the participant was unable to continue, even with encouragement.

### Standardization of the conditions for treadmill testing

Both validity samples (JIA and controls) performed the submaximal treadmill test prior to the maximal treadmill test on the same day, separated by approximately 30–60 min rest between each test. Both validity samples performed the submaximal treadmill test at 9.30 AM at the earliest, thereby most likely avoiding issues with morning stiffness. If unfamiliar with treadmill walking, participants practiced until they felt comfortable to start the submaximal treadmill test. The JIA reliability sample performed the submaximal treadmill test twice on the second test day after school, separated by approximately 15 min rest between each test. The same physiotherapist (KR) conducted all maximal and submaximal treadmill tests used to evaluate criterion validity and test-retest reliability. To test inter-rater reliability, KR and a second physiotherapist, both with more than 13 years of clinical experience in pediatric rheumatology, conducted the submaximal treadmill tests on the second test session.

### Statistical analyses

A power analysis was performed to estimate the required sample size for reliability testing of the submaximal treadmill test to achieve an ICC of 0.85 with a 95% confidence interval (CI) and an interval width of 0.2 (0.75 and 0.95). This calculation resulted in a sample size of 31 participants.

Descriptive data are presented as percentages, means (SD) and medians (25th–75th percentile) as appropriate. The COnsensus-based Standards for the selection of health Measurement INstruments (COSMIN) panel recommendations for measurement properties were followed for the evaluation of validity and reliability [17]. The observed VO_2peak_ from the maximal treadmill test was considered the criterion measurement.

Paired t tests were used to examine potential differences between the observed and estimated VO_2peak_ and between the estimated VO_2peak_ values from the three submaximal treadmill tests. Criterion validity and reliability were evaluated with two-ways mixed interclass correlation coefficient_agreement_ (ICC). ICC > 0.70 was considered acceptable [17]. Limits of agreement (LoA) (Bland and Altman method), standard error of measurement (SEM_agreement_) and smallest detectable change (SDC_95_) were calculated to evaluate the measurement errors of the submaximal treadmill test. The SEM_agreement_ represents the standard deviation of repeated measures in one patient, and was calculated with values from a two-way ANOVA. The SDC represents the minimal change that a patient must show on the scale to ensure that the observed change is real and larger than the measurement error. The SDC was calculated as 1.96 × √2 × SEM_agreement_ to obtain 95% CI. The SDC values at the group level (SDC_group_) were calculated as 1.96 × √2 × SEM_agreement_/√n.

The Bland and Altman method was used to assess whether there was any systematic disagreement between the submaximal and maximal treadmill test and between the submaximal treadmill tests for both test-retest reliability and inter-rater reliability through a Bland and Altman plot. LoA were calculated as the mean difference in scores ± (1.96 × SD of the difference).

Differences between patients and controls were tested with independent sample t tests and correlations with Spearman’s rho correlation coefficients.

All statistical analyses were conducted using SPSS version 23 for windows package (SPSS, Chicago, IL, USA) with the level of significance set at *P* < 0.05.

## Results

### Characteristics of patients and controls

**Fig. 1:**
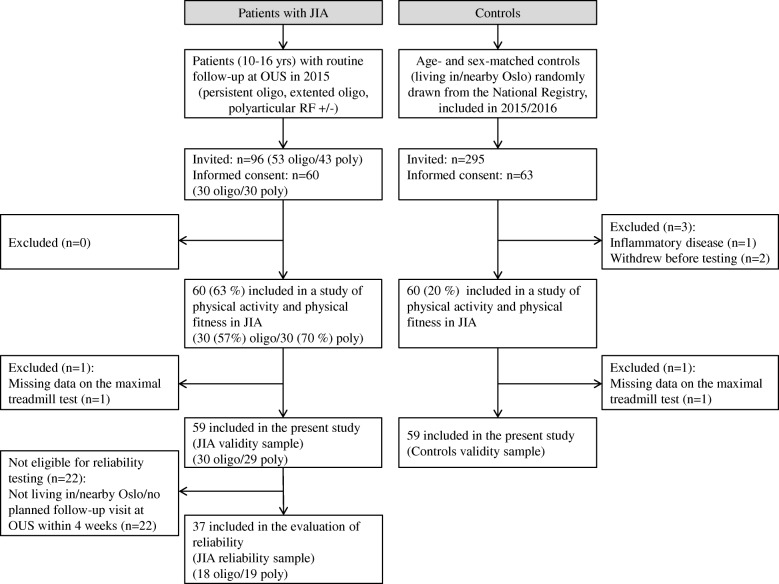
Flowchart over participant inclusion. *JIA* juvenile idiopathic arthritis, *OUS* Oslo University Hospital, *RF* rheumatoid factor

The flow of study participants is shown in Fig. 1. Demographic characteristics of the validity samples of patients and controls and the JIA reliability sample are displayed in Table 1. A total of 59 patients (50 girls) with oligo- (*n* = 30) and polyarticular (*n* = 29) JIA and 59 matched controls with complete data on the maximal and submaximal treadmill tests were included in the analyses to evaluate the criterion validity. Mean age (SD) was 13.6 (2.2) years in patients and 13.5 (2.6) years in controls. In patients, disease activity was moderate with a median (25th -75th percentile) JADAS of 3.2 (1.1–4.8), and 42% used biologic DMARDs (Table 1). Twenty-nine patients reported morning stiffness, but morning stiffness lasting 60–120 min or > 120 min was only reported by four patients and one patient, respectively. There was no clinical indication of cardiopulmonary side effects from synthetic or biologic DMARDs considered to be of importance for CRF. The JIA reliability sample included 37 patients (30 girls).

**Table 1:** Characteristics of patients with JIA and controls

	Patients with JIA validity sample (*n* = 59)	Patients with JIA reliability sample (*n* = 37)	Controls validity sample (*n* = 59)
Age (yrs)	13.6 (2.2)	13.6 (2.1)	13.5 (2.6)
Female sex, n (%)	50 (85)	30 (81)	50 (85)
Height (cm)	157.6 (12.5)	158.5 (13.0)	160.8 (12.3)
Weight (kg)	48.3 (11.8)	49.5 (12.7)	53.1 (15.2)
BMI (kg/m^2^)	19.2 (3.0)	19.4 (3.3)	20.1 (3.5)
Waist circumference (cm)	70.5 (9.8)	70.2 (8.9)	69.3 (9.2)
Pubertal status (pre-, mid-, and postpubertal %)	24/61/15	22/65/13	17/68/15
NRS current pain (0–10), n (%) score > 0	23 (38)	16 (43)	18 (30)
NRS pain previous week (0–10)	1.0 (0.0–3.0)	1.0 (1.0–3.5)	1.0 (0.0–3.0)
NRS fatigue previous week (0–10)	3 (2.0–6.0)	3.0 (2.0–6.0)	3.0 (1.0–5.0)
Oligo/poly, n (%)	30 (51) / 29 (49)	18 (49) / 19 (51)	NA
Disease duration (yrs)	7.5 (3.8)	7.5 (3.9)	NA
JADAS 71 (0–101)	3.2 (1.1–4.8)	3.0 (1.0–4.7)	NA
CHAQ score (0–3)	0.0 (0.0–0.3)	0.1 (0.0–0.4)	NA
Off medication, n (%)	12 (20)	9 (24)	NA
Synthetic DMARDs, n (%)	39 (66)	24 (65)	NA
Biologic DMARDs, n (%)	25 (42)	15 (41)	NA
Active disease, n (%)	20 (34)	12 (32)	NA
Inactive disease, n (%)	39 (66)	25 (68)	NA

Numbers are mean (SD) or median (25th – 75th percentile) unless otherwise indicated. *JIA* juvenile idiopathic arthritis, *BMI* body mass index, *NRS* numeric rating scale, *JADAS* juvenile arthritis disease activity score, *CHAQ* childhood health assessment questionnaire, *DMARDs* disease modifying anti-rheumatic drugs, *NA* not applicable

### Criterion validity in patients and controls

All participants were able to perform both the submaximal and maximal treadmill tests according to the test protocols for each test. None of the study participants experienced any adverse events during the treadmill testing. The results from the maximal and submaximal treadmill tests are shown in Table 2. For the maximal treadmill test, the mean HR_peak_, RER and RPE (Borg scale_6–20_) indicate that the participants exercised at their maximal capacity. This is underlined by the fact that all participants reported exhaustion as the reason for terminating the maximal treadmill test. As previously published [7], there were no significant differences between patients and controls for any variables from the maximal treadmill test. For the submaximal treadmill test, the HR and RPE reported during and immediately after the test indicate that both patients and controls exercised at submaximal intensity. In total, 44 (75%) patients and 41 (70%) controls reached the target HR of 70% of predicted HR_peak_ during the submaximal treadmill test. The remaining patients and controls reached a HR between 60 and 70% of predicted HR_peak_.

**Table 2:** Data characteristics of the submaximal and maximal tests used for the evaluation of criterion validity in patients with JIA and controls

		Patients with JIA (*n* = 59)	Controls (*n* = 59)
Maximal test			
VO_2peak_ (mL∙kg^−1^∙min^−1^)		45.1 (8.5)	46.5 (8.5)
Running distance (m)		909 (236)	968 (190)
Peak HR (beats/min)		196 (9)	197 (7)
Borg _6–20_		18.9 (1.9)	18.5 (1.0)
Respiratory exchange ratio		1.27 (0.12)	1.23 (0.10)
Test time (sec)		527 (99)	554 (76)
Speed (km/h)		8.3 (0.9)	8.5 (0.7)
Gradient (%)		11.5 (1.7)	11.8 (1.4)
Submaximal test			
Estimated VO_2peak_(mL∙kg^−1^∙min^−1^)		43.6 (9.9)	44.6 (7.9)
Walking distance (m)		751 (91)	739 (67)
Speed (km/h)		6.2 (0.8)	6.2 (0.6)
3 min	HR (beats/min)	134 (9)	132 (9)
	Borg _6–20_	9.6 (2.0)	9.4 (2.2)
8 min	HR (beats/min)	163 (14)	159 (14)
	Borg _6–20_	13.0 (2.2)	12.4 (2.2)

Numbers are mean (SD). *JIA* juvenile idiopathic arthritis, *HR* heart rate, *VO*_*2peak*_ peak oxygen uptake

**Fig. 2:**
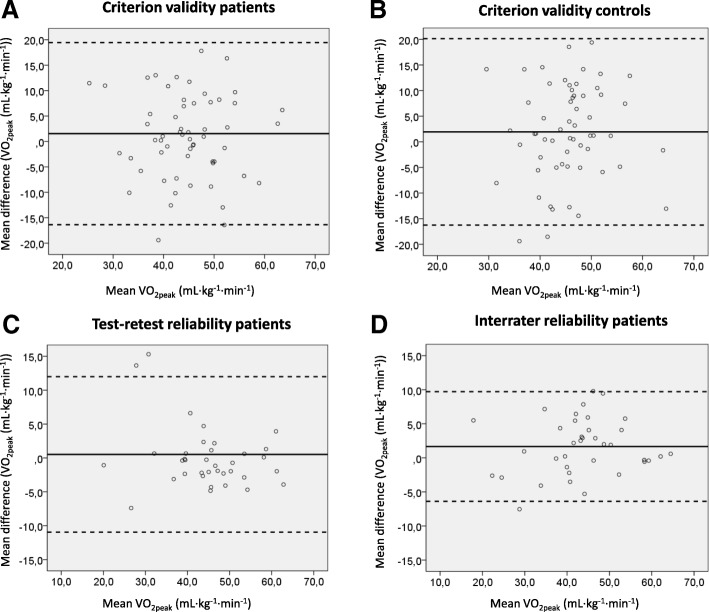
Bland and Altman plots with the mean scores [(observed+estimated VO_2peak_)/2] on the x-axis and mean difference between scores (observed-estimated VO_2peak_) on the y-axis for criterion validity in patients with JIA (**a**) and controls (**b**), and the mean scores [(test+retest)/2 and (Tester 1 + Tester 2)/2] on the x-axis and mean difference between scores [(test-retest) and (Tester 1-Tester 2)] on the y-axis for test-retest (**c**) and inter-rater reliability (**d**) in patients with JIA, respectively. *JIA* juvenile idiopathic arthritis, *VO*_*2peak*_ peak oxygen uptake

In patients, no significant difference was found between the observed and estimated VO_2peak_ (mL∙kg^− 1^∙min^− 1^); 44.8 (8.8) vs 43.2 (10.3), respectively, *P* = 0.18. The ICC (95% CI) at group level was acceptable; 0.71 (0.51, 0.82), while the single ICC value at individual level between the observed and estimated VO_2peak_ was not acceptable; 0.55 (0.34, 0.70). LoA showed large variation between the observed and estimated VO_2peak_ (− 16.4 to 19.4 mL∙kg^− 1^∙min^− 1^), with no systematic bias (Fig. 2a).

In controls, no significant difference was found between the observed and estimated VO_2peak_ (mL∙kg^− 1^∙min^− 1^); 46.53 (8.47) vs 44.60 (7.92), respectively, *P* = 0.12. Neither the ICC (95% CI) value at group level nor individual level were acceptable; 0.52 (0.21, 0.71) and 0.35 (0.11, 0.56), respectively. LoA showed large variation between the observed and estimated VO_2peak_ (− 16.3 to 20.1 mL∙kg^− 1^∙min^− 1^), with no systematic bias (Fig. 2b).

### Reliability in patients

Paired t tests showed no significant differences in estimated VO_2peak_ (mL∙kg^− 1^∙min^− 1^) when comparing the results from the submaximal treadmill tests (Table 3). Both the test-retest reliability and inter-rater reliability were acceptable at group level (ICC (95% CI) 0.91 (0.83, 0.96) and 0.96 (0.91, 0.98), respectively) and at individual level (0.84 (0.71, 0.91) and 0.92 (0.83, 0.96), respectively). The measurement errors were large for both test-retest reliability and inter-rater reliability (Table 3). The SDC_95_ values indicate that a change greater than 11.4 mL∙kg^− 1^∙min^− 1^ for the test-retest reliability and 8.6 mL∙kg^− 1^∙min^− 1^ for the inter-rater reliability would be required to be 95% certain that a change would not be the result of measurement error, but of a real change. The SDC_95group_ (at group level) values indicated that a change of greater than 1.5 mL∙kg^− 1^∙min^− 1^ for the test-retest reliability and 1.1 mL∙kg^− 1^∙min^− 1^ for the inter-rater reliability, would be required to be 95% certain that a change would not be the result of measurement error, but of a real change.

**Table 3:** Reliability and measurement error of the submaximal treadmill test in patients with JIA

	Test*	Retest*	Difference*	SEM_agreement_	SDC_95_	SDC_95group_
Est VO_2peak_ (mL∙kg^−1^∙min^− 1^)	44.9 (9.4)	44.3 (11.0)	0.5 (5.9)	4.1	11.4	1.5
	Tester 1*	Tester 2*	Difference*	SEM_agreement_	SDC_95_	SDC_95group_
Est VO_2peak_ (mL∙kg^−1^∙min^− 1^)	44.3 (11.0)	42.7 (10.5)	1.6 (4.1)	3.1	8.6	1.1

*Values are mean (SD). *JIA* juvenile idiopathic arthritis, *SEM* standard error of measurement, *SDC* smallest detectable change, *Est* estimated, *VO*_*2peak*_ peak oxygen uptake

*N* = 37

The Bland and Altman plots showed no systematic differences, but the LoA confirmed the large variability of agreement in estimated VO_2peak_ for both test-retest reliability and inter-rater reliability (Fig. 2c and d).

### Estimated VO_2peak_ and performance of the submaximal treadmill test between patients and controls, and correlation with disease variables

Estimated VO_2peak_ (mL∙kg^− 1^∙min^− 1^) and performance of the submaximal treadmill test did not differ significantly between patients and controls, all P’s > 0.15 (Table 2). Estimated VO_2peak_ (mL∙kg^− 1^∙min^− 1^) and performance of the submaximal treadmill test were also comparable between patients with persistent oligo- and polyarticular JIA and between patients with active and clinical inactive disease (data not shown). In patients, there were no correlations between any disease variables and estimated VO_2peak_ or walking distance (Table 4).

**Table 4:** Correlation between disease variables and estimated VO_2peak_ and walking distance in patients with JIA

Disease variable	Estimated VO_2peak_	*p*-value	Walking distance	*p*-value
Use of any medication	−0.07	0.59	−0.10	0.47
Use of synthetic DMARDs	0.04	0.79	−0.04	0.76
Use of biologic DMARDs	−0.03	0.80	0.02	0.81
JADAS 71 (0–101)	−0.03	0.84	0.08	0.54
CHAQ (0–3)	−0.13	0.35	−0.18	0.17
Active joints	0.10	0.48	0.21	0.16
Active joints in the lower extremities	0.09	0.46	0.20	0.13
Disease duration (years)	−0.13	0.34	0.00	0.97
Pain, current (NRS 0–10)	−0.21	0.11	−0.13	0.31
Pain, previous week (NRS 0–10)	−0.16	0.24	−0.02	0.88
Fatigue, previous week (NRS 0–10)	−0.07	0.58	−0.02	0.87

*JIA* juvenile idiopathic arthritis, *DMARDs* disease modifying anti-rheumatic drugs, *JADAS* juvenile arthritis disease activity score, *CHAQ* childhood health assessment questionnaire, *NRS* numeric rating scale, *VO*_*2peak*_ peak oxygen uptake

## Discussion

This is the first study to examine criterion validity and reliability of the eight-minute submaximal treadmill test aiming to estimate VO_2peak_ in patients with JIA. The results showed acceptable measurement properties on group level for both validity and reliability. The reliability was acceptable measured by ICC, but the measurement errors were large. On individual level, the validity was not acceptable, with large limits of agreement, and with no systematic bias. In controls, the validity of the submaximal treadmill test was not acceptable neither on group nor individual level. Patients with JIA and controls had similar estimated VO_2peak_ and submaximal treadmill test performance, and we found no associations with disease variables.

Compared to our results, studies on healthy adults [14] and women with rheumatic diseases [13] showed better validity. However, these studies applied different statistical methods than ours, making comparisons challenging. We applied ICC and Bland and Altman plots to evaluate criterion validity and reliability, statistical analyses methods recommended for these purposes by the COSMIN panel [17].

In the original study of the submaximal treadmill test, Ebbeling et al. [12] reported that there were no significant differences between the estimated and observed VO_2peak_ values in healthy adults, suggesting that the test has good predictive validity. We found similar results in both patients with JIA and controls when comparing estimated and observed VO_2peak_ mean values using paired t tests. The ICC value for evaluation of criterion validity at group level was acceptable in patients, but not in controls. However, our agreement analyses in both patients and controls showed large variation between the observed and estimated VO_2peak_, but with no systematic differences between the observed and estimated VO_2peak_. Agreement analyses were not conducted in the original article [12] or in other studies [13, 14]. However, a study of healthy adults [14] has reported a systematic overestimation of VO_2peak_ by 3.5 mL∙kg^− 1^∙min^− 1^ when testing at the moderate intensity (70% of the predicted HR_peak_) and an underestimation of VO_2peak_ by 3.5 mL∙kg^− 1^∙min^− 1^,when testing at the low intensity (50% of the predicted HR_peak_). The authors therefore suggested that if the purpose of using the submaximal treadmill test is to evaluate changes in CRF, all test sessions for the individual should be conducted at the same HR rather than the same speed. Thus, we aimed to test the participants at the same HR intensity (close to 70% of predicted HR_peak_) when conducting the submaximal tests. With this approach, the SDC was large for both test-retest and inter-rater reliability in our patients.

At group level, a change of more than 1.5 mL∙kg^− 1^∙min^− 1^ and 1.1 mL∙kg^− 1^∙min^− 1^ would be required to be 95% certain that a real change has occurred for test-retest- and inter-rater testing, respectively. These small SDC_group_ values suggest that the submaximal treadmill test is reliable on group level in patients, which is important for research purposes. When mean scores of a group of patients are used instead of individual patient scores, the measurement error becomes smaller and subsequently, the measure is more reliable [17]. If the submaximal treadmill test is used for evaluating change in individual patients in clinical settings, the large measurement errors must be taken into consideration. Specifically, a change of more than 11.4 mL∙kg^− 1^∙min^− 1^ and 8.6 mL∙kg^− 1^∙min^− 1^ are required to be 95% certain that a real change in a single individual has occurred, for test-retest- and inter-rater testing, respectively.

Submaximal tests are based on the assumption that there is a linear relationship between HR, oxygen consumption, and exercise intensity [8]. Therefore, an accurate age-predicted HR_peak_ is of importance. We used the same prediction of HR_peak_ as Ebbeling et al. [12] when they developed the test. This equation is proposed to underestimate HR_peak_ with increasing age and other equations in adult populations have been suggested [25, 26]. In our study participants, the mean predicted HR_peak_ was 207 in both patients and controls, while the mean HR_peak_ observed from the maximal treadmill test was 196 and 197 beat·min^− 1^ in patients and controls, respectively, suggesting overestimation of the predicted HR_peak_ when using the 220- age formula. In particular, as HR_peak_ varies between individuals, children with low HR_peak_ have probably been exercising at higher intensities than 70%. Importantly, there are many factors that may affect HR (e.g. hydration, caffeine, pain and anxiety). Nevertheless, the RPE and HR during the submaximal test indicate that the submaximal treadmill test is a test of submaximal intensity in these participants at group level. The formula by Tanaka et al. (208–0.7 x age) [25] was better for predicting HR_peak_ in both patients and controls than the formula by Nes et al. (211–0.64 x age) [26] and the 220-age formula [23] (data not shown). The formula by Tanaka et al. was also preferable over the 220-age formula in another study involving children [27].

The estimated VO_2peak_ and the performance of the submaximal treadmill test were comparable between patients with JIA and controls. This is in line with our previous findings studying the same cohort; directly measured VO_2peak_ was comparable between patients with JIA and controls [7]. Furthermore, we observed no correlation between disease variables and estimated VO_2peak_ and walking distance in patients. We have previously also reported that disease variables were not associated with any components of physical fitness in our patient cohort [7]. Taken together, our results suggest that disease variables are less important for physical fitness, including submaximal performance, in patients treated with a modern multidisciplinary management of JIA.

Our study has several strengths; we applied the COSMIN recommendations for evaluating the criterion validity and reliability of the submaximal treadmill test and the gold standard test was used as criterion measurement. Also, both physiotherapists conducting the submaximal tests for evaluation of reliability were experienced in pediatric rheumatology and one of these physiotherapists also conducted all maximal and submaximal treadmill tests used to evaluate the criterion validity. Also, the sample size was adequate. However, some limitations need to be considered. The equation to estimate the VO_2peak_ was developed in healthy adults aged 20–59 years, and it can be questioned if the formula is valid to use in patients with JIA and controls aged 10–16 years. Our JIA cohort seems well treated with low disease activity and functional disability, thus the findings may not be generalized to patients with higher disease activity or JIA categories not included in the current study. Additionally, the majority of the individuals included in the present study were females, which also could have hampered the generalizability of the results, although the formula used to estimate VO_2peak_ takes sex into account. Thus, future research should include other JIA categories and more males to improve the generalizability of the results.

## Conclusions

In patients with JIA, the submaximal treadmill test shows acceptable criterion validity at group level but not at individual level. The reliability of the test is acceptable, but with large measurement errors for both test-retest- and inter-rater reliability. Our results support that the submaximal treadmill test is valid and reliable for research purposes (on group level), but not optimal to estimate VO_2peak_ in individual patients. Estimated VO_2peak_ and performance of the submaximal treadmill test did not differ between patients and controls and were not associated with disease variables, probably reflecting the positive effect of modern multidisciplinary management of JIA.

## Abbreviations

6MWT: 6-min walk test
BMI: Body mass index
CHAQ: Childhood health assessment questionnaire
COSMIN: The COnsensus-based Standards for the selection of health Measurement INstruments
CPET: Cardiopulmonary exercise test
CRF: Cardiorespiratory fitness
DMARDs: Disease-modifying anti-rheumatic drugs
HR: Heart rate
HR_peak_: Peak heart rate
ICC: Interclass correlation coefficient
ILAR: International League of Associations for Rheumatology
JADAS: Juvenile arthritis disease activity score
JIA: Juvenile idiopathic arthritis
LoA: Limits of agreement
NRS: Numeric rating scale
OUS: Oslo University Hospital
RF: Rheumatoid factor
RPE: Rated perceived exertion
SDC: Smallest detectable change
SEM: Standard error of measurement
VO_2peak_: Peak oxygen uptake
